# Supplementary material for: The causal relationship between the human gut microbiota and pyogenic arthritis: a Mendelian randomization study
Source: Front Cell Infect Microbiol. 2024 Nov 26;14:1452480. doi: 10.3389/fcimb.2024.1452480 (PMC11629706; doi:10.3389/fcimb.2024.1452480)
Supplement: Supplementary file 1 [file DataSheet1.zip › mendelian test/ebi-a-GCST90027675.csv_leave_one_out.pdf]

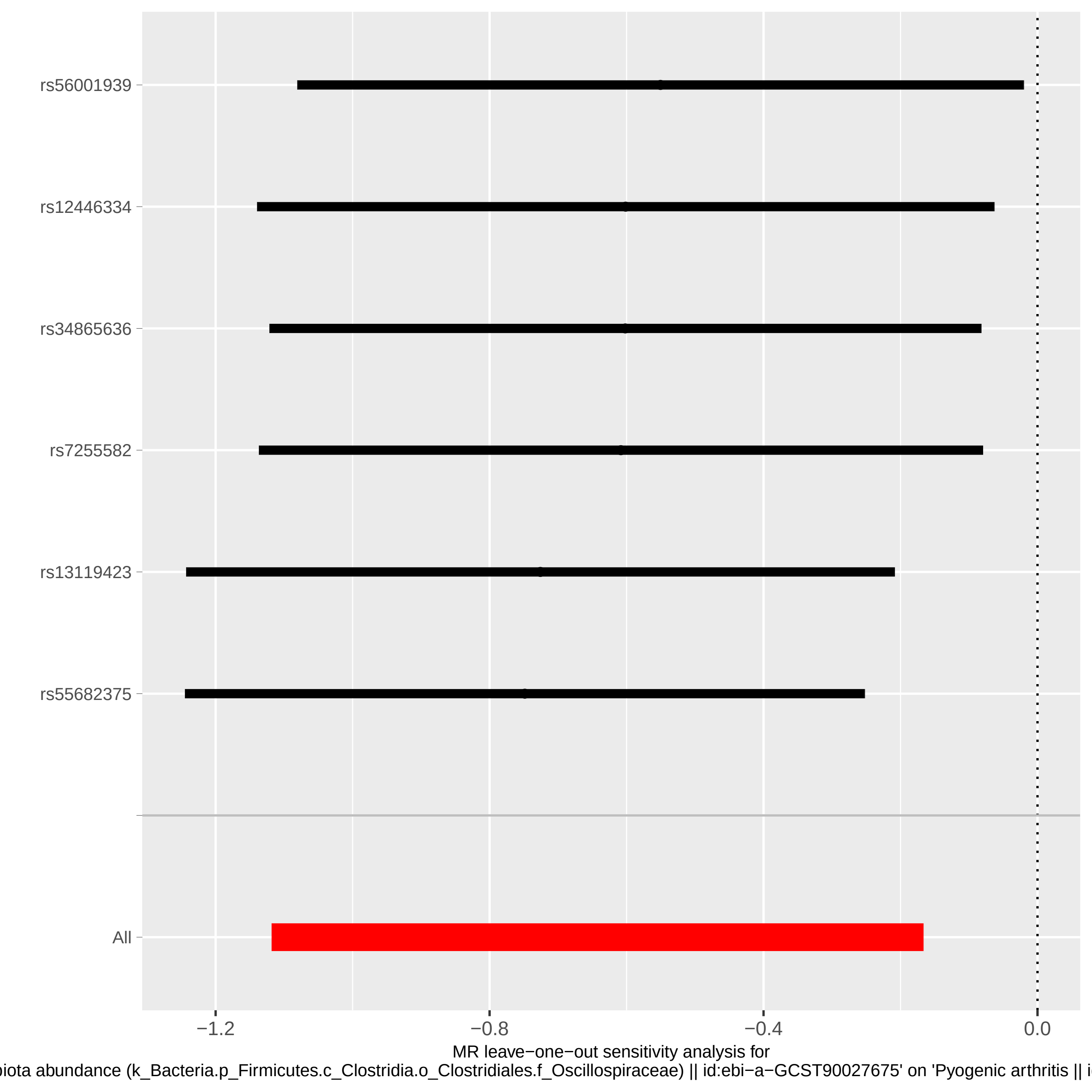

rs56001939
rs12446334
rs34865636
rs7255582
rs13119423
rs55682375
All
−1.2
−0.8
−0.4
0.0
MR leave−one−out sensitivity analysis for
biota abundance (k_Bacteria.p_Firmicutes.c_Clostridia.o_Clostridiales.f_Oscillospiraceae) || id:ebi−a−GCST90027675' on 'Pyogenic arthritis || i
